# How river drying influences greenhouse gas emissions: insights from species and gene shifts

**DOI:** 10.1093/ismeco/ycaf187

**Published:** 2025-10-16

**Authors:** Chaoran Li, Jun Hou, Thibault Datry, Tanveer M Adyel, Wei Zhou, Jun Wu, Guoxiang You, Tao Jin, Ye Deng, Lingzhan Miao

**Affiliations:** Key Laboratory of Integrated Regulation and Resources Development on Shallow Lakes, Ministry of Education, College of Environment, Hohai University, Nanjing, 210098, China; Key Laboratory of Integrated Regulation and Resources Development on Shallow Lakes, Ministry of Education, College of Environment, Hohai University, Nanjing, 210098, China; INRAE, UR Riverly, Center Lyon-Grenoble Auvergne-Rhône-Alpes, 5 Rue de La Doua CS70077, Villeurbanne Cedex, 69626, France; Centre for Nature Positive Solutions, Department of Biology, School of Science, RMIT University, Melbourne, VIC 3000, Australia; College of Geography and Remote Sensing, Hohai University, Nanjing, 210098, China; Key Laboratory of Integrated Regulation and Resources Development on Shallow Lakes, Ministry of Education, College of Environment, Hohai University, Nanjing, 210098, China; Key Laboratory of Integrated Regulation and Resources Development on Shallow Lakes, Ministry of Education, College of Environment, Hohai University, Nanjing, 210098, China; Guangdong Magigene Biotechnol Co Ltd, Guangzhou, 510000, China; Key Laboratory of Environmental Biotechnology, Research Center for Eco-Environmental Sciences, Chinese Academy of Sciences, Beijing, 100085, China; Key Laboratory of Integrated Regulation and Resources Development on Shallow Lakes, Ministry of Education, College of Environment, Hohai University, Nanjing, 210098, China

**Keywords:** metagenomics, intermittent rivers, N_2_O, CO_2_, stream biofilms

## Abstract

Drying is threatening global river ecosystems due to climate change, altering community composition and function even upon flow resumption. This mesocosm study investigated the greenhouse gas emissions fluxes and underlying mechanisms from benthic habitats prone to 20–100 days of drying. Results show that CO_2_ and N_2_O emissions from biofilms did not increase when drying increased, due to the changes in functional communities and genes. Notable is the transformation of biofilm from carbon source to sink following prolonged drying (mean emission fluxes ranged from 804.78 to −305.55 mg m^2^ h^2^). This was mainly due to strong increases in the abundance of genes involved in the Calvin–Benson–Bassham cycle (2.82 × 10^−5^ to 7.12 × 10^−5^), and functional taxa such as *gemmatimonadota* and *pseudomonadota*. These findings reveal a potential mitigation effect of drying on greenhouse gas emissions from rivers and streams, which could be relevant in the face of climate change.

## Introduction

Global warming has significantly increased the occupancy and the drying duration of intermittent rivers and ephemeral streams (IRES), defined as the river that cease flow and/or dry at some time and space along their course [1]. The effects of drying or drought on the ecosystem services provided by IRES are complex and multifaceted. The process of drying can lead to a reduction in biological habitats, disruption of ecological networks, interference with nutrient cycling and sediment transport, and significant impacts on biotic migration and the ecological recovery of IRES. How greenhouse gas (GHG) emissions are affected is more important, as global warming and river droughts will be exacerbated by a synergistic interaction between GHG emissions and increased drying duration. In addition, the more critical uncertainty is whether rewetting after prolonged drying can offset the negative effects of drying or whether legacy effects of prolonged drying persist and continue to affect river ecosystems. Consequently, the unknown ecological effects caused by river wet-dry alternation seriously hinder the study of intermittent river ecosystems and threaten the environmental protection and restoration of such ecosystems.

Biofilms, which are highly active microbial aggregates, play a crucial role in river microbial communities structure and ecological functions. Their responses to external hydrological changes contribute to a better understanding of feedback mechanisms [2]. Previous research on the ecological responses of intermittent rivers has shown that biofilms transition from being carbon sources to carbon sinks after prolonged drying [3]. However, the mechanisms underlying this change remain unclear. In addition to carbon dioxide (CO_2_), the response patterns of two other GHG–methane (CH_4_) and nitrous oxide (N_2_O)- to the increasing duration of drying in IRES also require thorough investigation. Gaining a better understanding of the mechanisms behind these emissions is essential for unravelling the unique climatic feedback present in intermittent river ecosystems.

In previous studies, the interaction between microbial community structure and ecological functions in intermittent rivers has typically been established using a partial least squares path model (PLS-PM) to examine the driving factors behind changes in ecological function. For instance, Li *et al.* [4] found a more stable ecological network among eukaryotic microorganisms, whose biodiversity predominates the ecological functions after prolonged disconnection (drying of more than 60 days). The high elasticity of the algae community may facilitate the transformation of biofilms from carbon sources to carbon sinks [3]. However, the PLS-PM relies on numerical correlations, identifying only potential significant influences, and it cannot analyse changes in relevant manipulated genes along the GHG emission pathways. Metagenomic analysis can provide insights into these emission pathways [5]. More importantly, a binning analysis can reconstruct the complete genomes, allowing for a focus on key active species that drive GHG emissions and enabling a better understanding of the relationship between microbial community structure and ecological functions [6]. Previous studies discussing different taxa (e.g. fungi, algae, bacteria) in biofilms individually may have overlooked the interactions among species in ecological functions, as biofilm GHG emissions represent the integrated metabolic output of the entire biological community. Therefore, this study integrates multiple taxa for community composition analysis, combined with metagenomic sequencing, to comprehensively analyse the mechanisms by which increasing drying affect biofilm GHG emissions.

Compared to studying the ecosystem condition of rivers during drying periods, examining the legacy effects of drying history can provide essential theoretical support for enhancing ecosystem resilience and ecological resistance [7]. Therefore, we placed biofilms that were cultivated and matured in natural rivers into an artificially simulated river channel. A control group with a constant flow and two experimental groups with water resumption after different drying cycles (20 and 100 days) were established. We identified major active microbial groups and genetic changes in the GHG pathways from the biofilms using high-throughput and metagenomic sequencing. To investigate the effect of drying intensification caused by global climate change on GHG emissions from intermittent streams, two questions must be addressed: (i) Is there mutual inhibition between CH_4_ and N_2_O with drying intensification, similar to CO_2_? (ii) What are the mechanisms by which drying exacerbates GHG emissions?

## Materials and methods

### Experimental design

Biofilms were inoculated from a natural river (The Qin Huai River, Eastern China) as per our previous study [8]. The study of biofilm colonization and water quality parameters are detailed in the Supporting Information (Text S1, Fig. S1, and Table S1). A control (constant flow), a short-term drying (SD) history (21 days of rewetting after 20 days of drying), and a long-term drying (LD) history (21 days of rewetting after 100 days of drying) (Fig. S1) were performed based on flowing artificial water channels in a greenhouse at 18 ± 2°C (constant water quality through the addition of nutrient solution; Table S2) [9]. For high-throughput sequencing and ecosystem function analysis, samples were collected from the experimental groups at five different time points (1, 3, 7, 14, and 21 days after rewetting), with four replicates for each time point, achieved by randomly selecting specific amounts of cobbles from the channels (*n* = 20). Additionally, samples were collected from the control group at the beginning and end of each rewetting period for the experimental groups (see Fig. S1). As for metagenome sequencing and biofilm structure, samples were collected at the end time of the rewetting process in both the experimental and control groups. Three replicates were obtained at each time point by randomly selecting specific quantities of cobbles from the channels (*n* = 3). More details are provided in the Supporting Information (Text S1 and Fig. S1).

### Measurement and imaging of biofilm structure and ecosystem functions

A fixed area of the biofilm (30 ± 2 cm^2^) was dried (105°C, 24 h) and calcined (450°C, 5 h) to determine the water content and ash-free dry weight, reflecting shifts in biofilm biomass [10]. The structure of the biofilms was visualized using multiple fluorescent staining techniques combined with a confocal laser scanning microscope [11]. Subsequent quantitative analysis of the different fluorescence signals was performed using LAS-X software.

GHG emission rates were measured using a static box-gas chromatography (Trace GC, ThemrmoFisher). The community respiration and gross primary productivity were calculated based on changes in dissolved oxygen in the static box. In addition, ecosystem functions related to the cycling of carbon and nitrogen were measured, including *α*-glucosidase, *β*-glucosidase, cellobiose hydrolase, glucokinase pentoses, leucine aminopeptidase, nicotinamide adenine dinucleotide, nitrate reductase, nitrite reductase, nitric oxide reductase, nitrous oxide reductase, an ammonia monooxygenase, hydroxylamine oxidoreductase, and nitrite oxidoreductase. More detailed experimental determinations were provided in the Supporting Information (Texts S2 and S3).

### High-throughput and meta-genome sequencing

To enable a comprehensive assessment of biodiversity and a targeted evaluation of genome-level responses across treatments, biofilm samples were subjected to both high-throughput amplicon sequencing and meta-genome sequencing. The timing of sample collection and experimental scheduling followed the design detailed in Fig. S1. Partial eukaryotic 18 s rDNA 528F (5′-GCGGTAATTCCAGCTCCAA) and 706R (5′-AATCCRAGAATTTCACCTCT) and bacterial 16 s rDNA 515F (5′-GTGCCAGCMGCCGCGGTAA-3′) and 806R (5′-GGACTACHVGGGTWTCTAAT-3′) genes were amplified at the V4 hypervariable regions. The details of MiSeq sequencing are also available in the Supporting Information Text S4. Metagenomic assembly of clean reads using MEGAHIT (v1.2.9, https://github.com/voutcn/megahit), specifying a minimum configuration size of 500 bp. Taxonomic classifications and functional annotations of non-redundant unigene sequences were made on NR, KEGG, CAZy, and other databases. Refer to the Supporting Information for detailed experimental determination and statistical analyses of meta-genome sequencing data (Text S5).

### Analyses of genome bins

To generate high-quality genomes, clean reads from each sample were co-assembled with metaSPAdes (v3.15.5). After co-assembly, contigs with lengths ≥2500 bp were clustered into meta-genome-assembled genomes (MAGs) using MetaBAT2. The taxonomy of MAGs was determined with GTDB-tk (v2.3.0). CheckM2 was employed to assess the completeness and contamination of the MAGs using the default parameters. MAGs with completeness ≥50% and contamination ≤10% were retained for downstream analysis. The replication activity of the MAGs was estimated using iRep (default settings), while relative abundance was determined with CoverM (https://github.com/wwood/CoverM). Metabolic reconstruction of each MAG was accomplished using METABOLIC, which integrated functional annotations from multiple databases, including Kyoto Encyclopedia of Genes and Genomes (KEGG), The Institute for Genomic Research protein families database (TIGRfam), Protein families database (Pfam), database for automated Carbohydrate-active enzyme ANnotation (dbCAN2), MEROPS (the peptidase database), and custom hidden Markov model database. METABOLIC also incorporates protein motif validation to identify proteins accurately and determines the presence or absence of metabolic pathways based on KEGG modules. The contribution of each microbial group to biogeochemical processes was calculated using METABOLIC-C.

### Statistical analyses

The significant differences in biofilm functions and biodiversity among groups were performed by a one-way analysis of variance (ANOVA) analysis after ensuring that all assumptions of ANOVA were met. This included verifying the independence of samples, testing for normality using the Shapiro–Wilk test, and confirming homogeneity of variances with Levene’s test. The data will be appropriately transformed (e.g. log-transformed) where necessary to meet these assumptions. The results were considered statistically significant at *P* < .05. PERMANOVA (Adonis) analysis [12] on the online platform (http://cloud.magigene.com/yomics) was used to test the differences in beta diversity between groups. Community heterogeneity was calculated through the dissimilarity between experimental communities based on the Bray–Curtis [13]. The similarity between the experimental group and the control based on the Bray–Curtis, was calculated to test community resilience [13]. The Mantel test was conducted based on Pearson’s correlation with the ggcor package in R [14]. Biofilm microbial community stability was evaluated by robustness to investigate the effects of biofilm structural characteristics and enzyme activities on biofilm GHG emissions and ecosystem metabolism. The detailed calculation formula is available in the Supporting Information. The calculation of other network topologies is also available in Supporting Information (Text S6).

## Results and discussion

### Increased biofilm fragmentation and biological activity with drying duration

**Figure 1:**
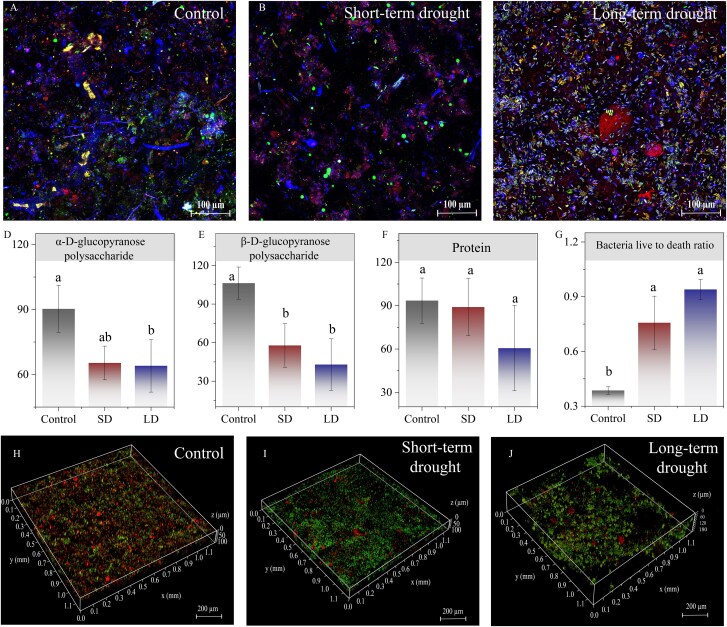
Laser scanning confocal microscope results of the three-dimensional structure of biofilms and the ratio of live to dead bacteria in biofilms. Three-dimensional structure of biofilm (A–C), three-dimensional parameters in LAS-X (D–F), live-to-death ratio of bacteria in biofilm (G), and the imaging of live and death bacteria (H–J). The letters a, b, and c over the standard error indicate significant differences between samples.

To visually investigate whether an increase in drying history affects the microstructure of biofilms, we quantitatively and qualitatively determined the three-dimensional structure of extracellular polymers (EPS) and the bacterial live-to-death ratio of biofilms (Fig. 1). The coping strategy with the drying of biofilm may be the contraction of EPS [2] and the inability to recover during rewetting [15], resulting in the fragmentation of biofilms after LD, and the excessive fragmentation might delay the recovery of ecosystem functioning [16].

With limited resources, biofilms may prioritize the use of carbon sources for more urgent metabolic activities rather than the production of polysaccharides, resulting in a significant decrease in polysaccharide content (alpha-D-glucopyranose polysaccharide and beta-D-glucopyranose polysaccharide) with increasing drying duration (Fig. 1D and E). In addition, polysaccharides act as adhesives in EPS [17], and the decrease in polysaccharides reduced the water retention capacity of EPS, providing a strong explanation for the increased fragmentation of biofilms after prolonged drying (Fig. 1A–C). There was no significant change in the protein content in the EPS composition (Fig. 1F), probably because proteins play an essential role in cellular defense [18], and there is a dynamic balance between degradation and production of proteins in response to hydrological changes outside the surface.

Some highly drought-resistant and highly resilient bacteria contained in the biofilm survived drying screening and rapidly colonized under rewetting conditions [2], resulting in a significantly higher proportion of live bacteria with increasing drying duration (Fig. 1G–J). The higher ratio of live bacteria implied an enhancement of the metabolic activity of the biofilm [19], such as organic matter degradation and nutrient cycling etc. However, the overpopulation of particular dominant flora may also decrease community diversity, potentially reducing functional diversity.

### Greenhouse gas emission of biofilms

**Figure 2:**
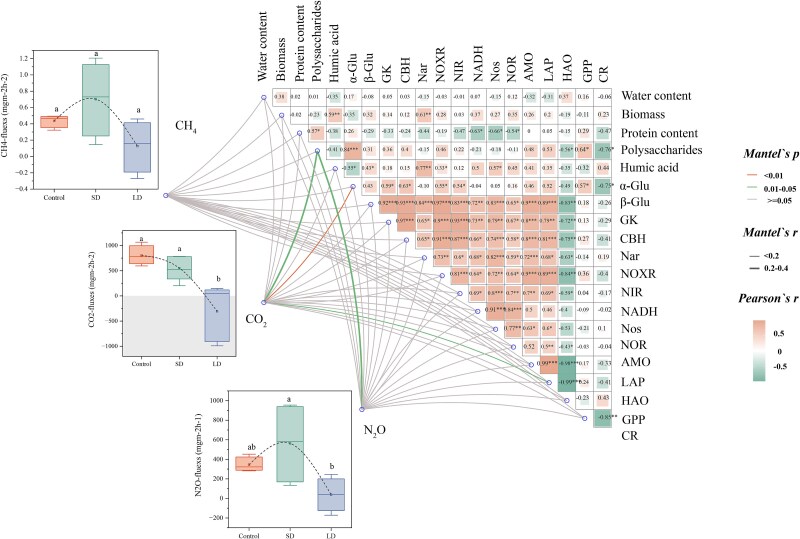
The change in biofilm GHG emission fluxes during rewetting after different drying histories and the mantel test of biofilm GHG emission fluxes with various structural and functional indicators associated with the carbon and nitrogen cycles. The letters a, b, and c over the standard error indicate significant differences between samples.

With increasing drying history, significant reductions in CO_2_ and N_2_O emissions were observed (Fig. 2). The content of polysaccharides showed a significant correlation with CO_2_ and N_2_O emission (Fig. 2), possibly because polysaccharides can bind to membrane proteins to regulate N_2_O synthesis [20]. These polysaccharides highlight the resource regulation strategy of biofilms in response to changes in IRES hydrology [21], where polysaccharides are used more for biofilm growth and metabolic recovery than for respiratory depletion to stabilize biofilm structure. In addition, *α*-glu exhibited a significant correlation with CO_2_ emission (Fig. 2), attributed to their involvement in cellulolytic catabolism and provided an essential substrate for biofilm respiration, thus contributing to CO_2_ emission [22]. As the aerobic environment in the artificially simulated river was unfavorable for CH_4_ production, resulting in low CH_4_ emission fluxes, the differences between different experimental groups in the results were not significant (Fig. 2).

In conclusion, there was a mutual inhibition between the emission of all three GHGs with the increase in river drying duration, which was conducive to restoring ecosystem dynamic balance. However, the activities of enzymes involved in carbon and nitrogen metabolism were not significantly correlated with GHG emissions, and further investigations at the species and gene levels are needed to analyse the mechanisms of GHG emissions.

### Changes in biodiversity of biofilm experiencing different drying histories

Habitat changes and resource scarcity in wet and dry alternations may be responsible for the significant decrease in biodiversity (*α* and *β* diversity) with increasing drying duration (Figs S2–S4 and Table S3). Community heterogeneity of biofilms showed a significant decrease after experiencing drying. However, it did not continue to decrease with increasing drying cycles (Fig. S4C), possibly due to the rapid screening of species by river drying [23]. Based on the legacy effect of river drying, the resilience of the biofilm community exhibited a significant decrease with increasing drying duration (Fig. S4D), which indicated that flow restoration alone could not achieve effective restoration of biofilm community status in intermittent streams.

**Figure 3:**
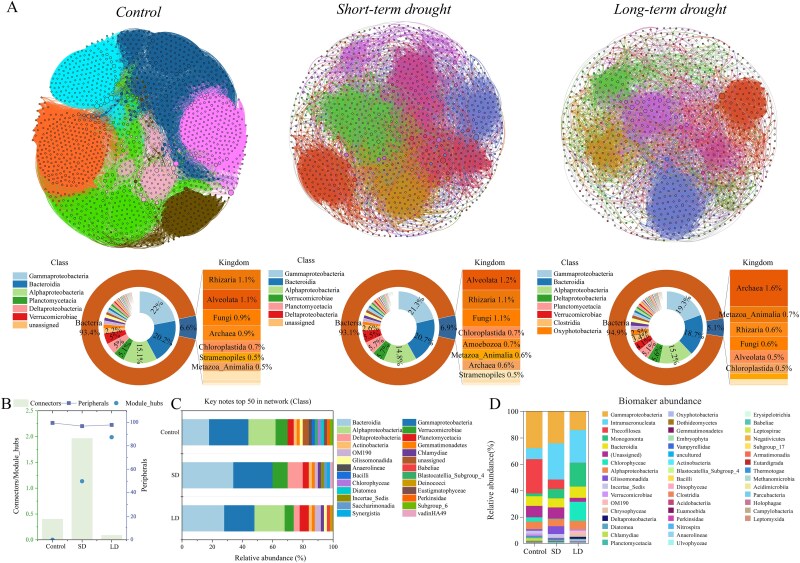
The results of network structure visualization of biofilm-integrated taxa and network species composition at class and kingdom level (A), network properties of key species (B), key species nodes within the network that play structurally important roles (C), and biomarker species composition among different treatment groups at class level (D).

Further analysis of the species composition of the microbial ecological network revealed that bacteria consistently dominated the community network, and the dominance of archaea significantly increased with increasing drying duration (Fig. 3A). Among them, the unique structure of the archaea, like ether-linked lipids in the cell membrane and phenotypic switching with environmental stresses, ensures that archaea are highly drought-tolerant [24, 25]. It also contributes to archaea’s rapid recovery and gradual dominance during rewetting after prolonged drying (Fig. 3A). More specifically, *gammaproteobacteria*, *bacteroidia*, and *alphaproteobacteria* consistently dominate the community network at the class level (Fig. 3A). The contribution of *deltaproteobacteria* gradually increased with increasing drying duration (Fig. 3A), resulting from the survival strategies in which *deltaproteobacteria* might release myxospores in unfavorable environments [26].

The ecological network topology results showed that the biofilm ecological network’s complexity (Avg K) decreased significantly with prolonged drying history (Fig. S5A). It might happen because the alternating wet and dry hydrological changes significantly reduced biodiversity (Fig. S4) and disrupted species interactions [27]. The cooperativity and tightness (GD; average path length of the ecological network) between species were enhanced considerably after wet and dry alternations (Fig. S5B and C). The implication is that tight and reciprocal network relationships contribute to better adaptation of microbial communities to environmental changes. Meanwhile, it also explained the significant increase in microbial network stability (Robustness) after prolonged drying (Fig. S5D), contributing to the long-term stability and functional diversity of the ecosystem [28].

### Changes in biofilm keystone flora with increasing intermittent duration

The proportion of nodes with module-hubs in the biofilm species interactions network increased with drying duration, indicating that microbial communities respond to external environmental pressures through a tighter modular structure (Fig. 3B and Fig. S6). The changes in connectors and the response of network structural robustness indicate that short-term alternations between wet and dry conditions enhance the stability of microbial networks (Fig. 3B and Fig. S5). In contrast, prolonged drying stress significantly reduces biodiversity, leading to a marked decline in stability [29]. Key species screening of the ecological network based on the betweenness centrality of nodes revealed that *bacteroidia* and *gammaproteobacteria* consistently played critical roles in the biofilm ecological network (Fig. 3C). Notably, the importance of *alphaproteobacteria* was only shown in control and after prolonged drying (Fig. 3C). On the other hand, *deltaproteobacteria* was the only necessary group after wet and dry alternations (Fig. 3C). The prevalence of many members of the *proteobacteria* can be attributed to their highly flexible metabolic pathways [30], which allow them to survive in extreme environments [31]. Rapid growth rates and metabolic responsiveness [32, 33] characterize *Gammaproteobacteria* and *alphaproteobacteria*. Drying may reduce the abundance of other microorganisms more dependent on stable aquatic environments. At the same time, these groups can rapidly consume newly available resources (e.g. dissolved organic matter, nutrients) during rewetting, facilitating rapid growth. These characteristics allow them to recover quickly and dominate the microbial community during rewetting phases following drying (Fig. 3D and Fig. S6). On the other hand, this dominance could reduce microbial community diversity, potentially affecting the ecosystem stability and ecosystem functions [32, 33]. In addition, *bacteroids* play a critical role in the decomposition of organic matter; their dominance could enhance carbon fixation and mineralization processes, affecting the overall carbon balance.

Finally, Linear Discriminant Analysis Effect Size (LEfSe) analyses of biofilm species composition at different stages revealed that most signature species showing significant differences between groups were concentrated in *gammaproteobacteria* and *intramacronucleata* (Fig. 3D and Fig. S7). Although these groups include a wide diversity of species and represent relatively broad taxonomic classifications, the observed shifts at the class level still offer meaningful ecological insights into community turnover during drying–rewetting cycles. The significant decrease of *thecofiosea* with increasing drying duration resulted from their heavy dependence on moisture (Fig. 3D). *Intramacronucleata*, *monogononta*, and *chlorophyceae* increased significantly after wet-dry alternation (Fig. 3D). Because of their role as photosynthetic organisms [34], *chlorophyceae* can use light energy to fix CO_2_, and the increased abundance of *chlorophyceae* directly increases the primary productivity of the ecosystem [35]. The substantial organic carbon produced by the photosynthesis of green algae is likely to be stored primarily in biofilms, gradually transforming these biofilms from carbon sources to carbon sinks (Fig. 2). As rotifers, *monogononta* obtains energy and nutrients primarily by consuming phytoplankton such as green algae, bacteria, and detritus [36, 37]. The increase in their abundance suggested that during the post-drying rewetting period, the nutrients available in the system (such as organic carbon produced by algal and bacterial communities) were sufficient to support higher trophic levels. With the increase in *chlorophyceae*, the carbon fixation capacity during rewetting is significantly increased. Consumers such as *monogononta* and *intramacronucleata* can further utilize the fixed organic carbon, with some of the carbon entering the food chain rather than being mineralized and released.

### Key active flora involved in greenhouse gas emissions

**Figure 4:**
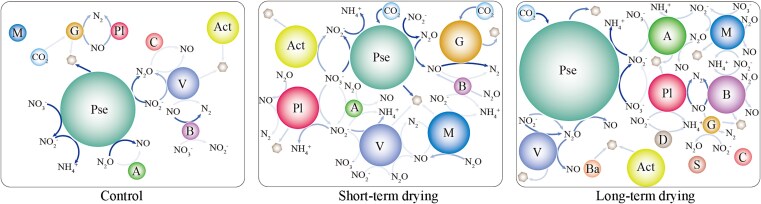
The results of binning analyses. The size of circle represents the bacteria’s relative abundance, and the shade of the arrow color represents the relative contribution to the process. Hexagon indicates fermentation intermediates (acetate, lactate, etc.).

Metagenomic binning analysis revealed that the dominant microbial communities involved in carbon fixation shifted from group *gemmatimonadota* to *pseudomonadota*, with the relative abundance of *pseudomonadota* increasing significantly as the drying duration extended (Fig. 4). Notably, *pseudomonadota* consistently dominated N_2_O uptake and emission processes. Following short-term wet-dry hydrological cycles, additional microbial taxa—including *planctomycetota*, *acidobacteriota*, *verrucomicrobiota*, *myxococcota*, and *bacteroidota*—became involved in N_2_O oxidation (Fig. 4), leading to a decrease in N_2_O production. Prolonged drying further enhanced N_2_O oxidation by *pseudomonadota*, accompanied by a significant rise in its abundance (Fig. 4). Moreover, other N_2_O oxidizing microbial groups also increased to varying degrees, resulting in a further reduction of N_2_O emissions (Fig. 4). *Bacteroidia* and *gammaproteobacteria* are not only key species in the ecological network of biofilm (Fig. 3) but also the main active microorganisms dominating N_2_O emission (Attachment 1). *Gammaproteobacteria* performed an important role in the initial reaction of biofilm denitrification, and *bacteroidia* mainly influenced the reduction of N_2_O to N_2_ (Attachment 1). *As a biomarker species among groups, Gammaproteobacteria* also played an important role in CO_2_ fixation, significantly increasing drying duration (Attachment 1). Meanwhile, *gammaproteobacteria* served as the main active group in CO_2_ production from biofilm fermentation (Attachment 1), which decreased with increasing drying duration, collectively explaining the biofilm transition mechanism from carbon source to carbon sink after prolonged wet-dry alternation. In addition, statistical analysis of all microbial communities involved in GHG-related carbon and nitrogen cycles revealed that more extended drying periods led to substantial enrichment of microbial groups associated with carbon fixation pathways (Fig. S9). Additionally, the microbial taxa involved in N_2_O production gradually decreased, while those participating in N_2_O oxidation increased significantly (Fig. S9), culminating in a marked reduction in N_2_O emissions.

The enrichment of carbon-fixing microbes, like *pseudomonadota*, suggests a potential for enhanced carbon sequestration in these ecosystems, which could help offset atmospheric CO_2_ levels and mitigate climate change impacts [38]. The significant reduction in N₂O emissions—a potent GHG—due to increased N_2_O oxidation by microbial communities further contributes to the potential mitigation of GHG effects [39]. Understanding these microbial shifts is essential for predicting how IRES respond to climate variability. Enhanced carbon fixation and reduced N_2_O emissions may improve the resilience of these ecosystems to environmental stresses, supporting their functional integrity and their role in global carbon and nitrogen cycles [40].

### Modification of carbon source utilization strategies promotes carbon sink in biofilms

**Figure 5:**
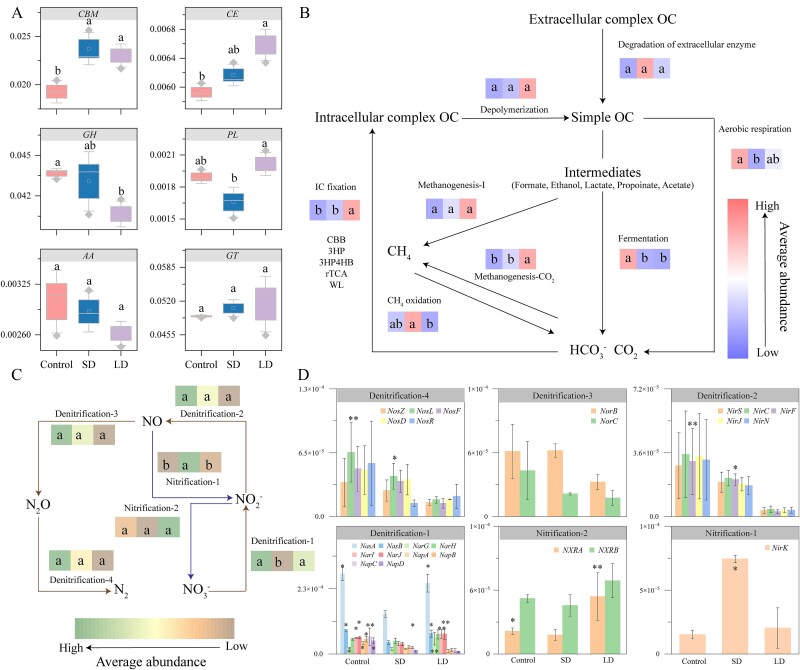
Metabolic potential of biofilms for different carbohydrates after different drying histories (A), and the core pathways in the carbon (B), and nitrogen (C–D) cycles based on the KEGG annotation of the gene sets.

Functional genes of carbohydrate-binding modules (CBMs) and carbohydrate esterase (CE) in biofilms increased significantly with increasing drying history (Fig. 5A), which is possibly due to the reduction in nutrient availability in biofilms during drying leading to a shift in microbial communities toward atypical carbon sources for survival [41, 42]. When flow recovery occurs, CBMs and CEs can decompose carbohydrates into many available small-molecule carbon sources for biofilm growth and storage [43]. For example, significant increases in CBM62 and CE1, which are biomarker genes in the LD group (Fig. S8A–B), enhance the efficiency of catalyzing the metabolism of complex carbohydrates (xyloglucan, cellulose, etc.), and speed up enzyme reactions to break down complex carbon sources into simpler ones [44]. Based on the decrease in the functional genes of polyglycoside hydrolases, small molecular carbon sources gradually accumulate in biofilm [45]. A consequent enhancement of the biofilm carbon sink will further increase the resilience of stream benthic ecosystems to environmental fluctuations [46].

The results showed that the functional genes of glycohydrolase (PLs) decreased after SD but increased significantly after increasing the historical duration of drying (Fig. 5A). This is because PLs, a high-energy functional gene, is also used to decompose complex sugars [47, 48]. After the SD, some non-essential or high-energy functional genes will decrease to better adapt to the short-term environmental pressure. There are only complex carbon sources that microorganisms can degrade when faced with an environment with insufficient carbon sources [49], such as the increase of PLs during rewetting after LD (Fig. 5A). The significant increase of AA1 and GT77 in the LD group’s biomarker genes reflects the organisms’ adaptive mechanism to the alternating dry and wet environments (Fig. 5A and Fig. S8A–B). The AA1 enzyme strengthens the structure of the cell wall through antioxidants. In contrast, the GT77 enzyme ensures the survival and reproduction of the organisms under drying conditions by enhancing the stability and water retention of the cell wall (Fig. S8A–B). The mechanism is also essential in carbon cycle of ecosystems, ensuring the long-term stability of carbon by maintaining the storage and balance of carbon resources.

The core pathways in the carbon cycles were mapped after KEGG annotation of the gene sets (Fig. 5B) [50] to identify the emission mechanisms of GHG after different drying histories. Consistent with the CAZy results, the metabolism of complex carbon sources was elevated, with intracellular complex carbon metabolism showing a particularly pronounced increase (Fig. 5B and Fig. S8C). We focused on three metabolic pathways for simple carbon sources: methanogenesis-1, fermentation, and aerobic respiration. Both fermentation and aerobic respiration decreased significantly with increasing drying duration, whereas methanogenesis exhibited an upward trend (Fig. 5B and Fig. S8C). Moreover, carbon fixation increased significantly as the drying duration extended (Fig. 5B and Fig. S8C), causing the biofilm to shift from a carbon source to a carbon sink under prolonged drying conditions. Among the five common carbon fixation pathways, genes associated with the Calvin–Benson–Bassham (CBB) cycle had the highest abundance and increased significantly with longer drying durations (Fig. 4B and Fig. S8C–D). In contrast, the other carbon fixation pathways fluctuated at lower levels. Enhanced carbon fixation during rewetting periods can mitigate atmospheric CO_2_ levels by acting as temporary carbon sinks, potentially buffering the effects of climate change [38]. This process influences internal carbon dynamics within aquatic ecosystems, affecting nutrient availability, and microbial community structures [51]. This enhanced carbon sink function may improve the resilience of intermittent river ecosystems to environmental stresses by promoting carbon storage and supporting essential microbial processes during ecosystem recovery after drying [52].

Although methanogenesis-related genes were enriched after long drying, several rate-limiting constraints can suppress net CH₄ production or enhance its removal upon rewetting (Fig. 5B, Fig. 1, and Fig. S8C). Gene or pathway abundance does not necessarily translate into *in situ* process rates under substrate and thermodynamic limitations; prolonged desiccation can deplete labile carbon and shift methanogenesis toward hydrogenotrophic routes that are more sensitive to H₂ availability and electron-acceptor competition, thereby decoupling genomic potential from realized methanogenesis [53]. Alternative electron acceptors (e.g. NO₃^−^/NO₂^−^, Fe(III), N₂O) that accumulate or become more available across drying–rewetting can inhibit methanogenesis and divert common substrates, further constraining CH₄ production despite the presence of methanogenesis genes [54, 55]. Rewetting also reintroduces oxygen into surface biofilms, deepening O₂ penetration and enabling rapid recovery of methanotrophic activity; even where methanotroph abundance has been reduced, oxidation can be resilient and offset increases in generation potential [56, 57]. Moreover, metagenomic signals represent genetic potential rather than realized activity: increases in methanogenesis or methane-oxidation gene inventories may not scale with transcription or enzyme turnover, and multi-omics studies commonly report partial decoupling between DNA-level abundance and RNA/protein-level activity in methane-cycling guilds [58, 59]. Expression of key enzymes is strongly regulated by environmental conditions and micronutrients, so the net CH₄ flux reflects a shifting balance between constrained production and efficiently recovering oxidation under fluctuating redox and resource conditions, explaining why pathway enrichment under long drying does not necessarily yield higher emissions [60].

These findings highlighted the critical role of microbial processes in biofilms of intermittent rivers in regulating carbon cycling and GHG emissions [40], for example, by adopting a more flexible nutrient utilization strategy (shifting from metabolizing simple to complex carbon sources). Understanding these processes is crucial for predicting IRES ecosystem responses to climate variability, particularly with the increasing frequency and intensity of drying due to global climate change [61, 62].

### Responses of biofilm nitrogen cycle path gene during rewetting to different drying histories

The core associated with N_2_O emissions involves nitrification and denitrification (Fig. 5C). Functional genes that play a pivotal role in the initial step of denitrification—specifically NasB, NarG, NarH, NarI, and NarJ—showed a significant decrease after short-term wet-dry alternation. The considerable increase following prolonged drying promotes the conversion of nitrate to nitrite (Fig. 5C). In contrast, most functional genes governing the subsequent steps of the denitrification process demonstrated a decreasing trend with extended drying duration (Fig. 5C). This suggests that after drying, the denitrification process may be partially inhibited, especially in the subsequent conversion steps, which would reduce N₂O production. In addition, NXRA and NXRB, which are involved in the last step of the nitrate reduction process, gradually increased with drying duration (Fig. 5B), accelerating the conversion of nitrate (${\mathrm{NO}}_3^{\hbox{-} }-\mathrm{N}$) to nitrite (${\mathrm{NO}}_2^{-}-\mathrm{N}$) and achieving a dynamic balance in N cycle, which further reduces the accumulation of nitrite that can be used in the denitrification process to produce N₂O. Meanwhile, NirK (conversion of N₂O to NO) was significantly reduced after prolonged drying (Fig. 5B), similarly reducing nitrite production (a source of substrate for N₂O production during denitrification) [63, 64]. Due to the dynamic adjustment of denitrification and nitrification processes under dry conditions, biofilms may gradually change from a traditional source of N₂O emissions to a more stable nitrogen cycler [65, 66].

## Limitations and environmental implications

While this study provides significant insights into the impact of LD on biofilm functions and GHG emissions in IRES, several limitations should be considered. First, the artificial river channel experiments, although meticulously designed, may not fully replicate the complex interactions present in natural river systems. The controlled environment may limit the generalizability of the results to more variable and diverse natural settings, like the changing temperature, elevation, and vegetation coverage on rivers. Additionally, the study focused on specific drying durations and rewetting periods, which may not capture the full spectrum of hydrological variability experienced in different climatic zones. The fact that some low-abundance algal species or functions may have been overlooked during the metagenomic sequencing process may lead to an incomplete understanding of ecosystem responses to drying. The interpretation of LEfSe results was constrained by the resolution limits of the sequencing approach, which classified most indicator taxa at the class level. For instance, *Gammaproteobacteria* and *Intramacronucleata* encompass a broad range of phylogenetically and functionally diverse organisms, and finer taxonomic or ecological patterns may have been overlooked. This limitation stems from the sequencing depth and read length used, which did not support accurate genus- or species-level assignments across all samples. Future research should incorporate higher-resolution approaches such as full-length 16S rRNA sequencing to enhance taxonomic resolution, and metatranscriptomics or metabolomics to improve functional inference beyond genomic potential. While metagenomics identifies which genes and taxa are present, it does not capture their transcriptional activity or dynamic interactions. By integrating gene expression profiles and metabolic outputs, future studies could uncover how key microbial groups and functional genes are actively involved—and potentially coordinated—in regulating carbon metabolism and other ecosystem functions. Such multi-omics strategies will provide a more comprehensive understanding of not only who is present, but also what they are doing and how they interact, ultimately enabling more accurate predictions of biofilm responses to drying–rewetting cycles.

The findings from this study have profound environmental implications, particularly in the context of global climate change. The observed shift from biofilms acting as carbon sources to carbon sinks following prolonged drying suggests that IRES could significantly regulate carbon balance in freshwater ecosystems. This transformation could mitigate some of the carbon losses associated with drought, potentially influencing global carbon cycling. However, the reduction in GHG emissions, such as CO_2_, CH_4_, and N_2_O, could have complex feedback effects on climate systems. Concurrently, drought conditions could diminish the pollutant removal capacity of biofilm, potentially altering the self-purification capacity of rivers and impacting overall ecosystem health. The adaptive responses of microbial communities, including the restructuring of metabolic functions and the increase in autotrophic organisms, highlight the resilience of these systems but also raise concerns about the long-term sustainability of these ecosystems under persistent drying conditions. Understanding these dynamics is crucial for developing strategies to protect and manage IRES, particularly as they become more prevalent due to climate change.

## Conclusion

As a result of climate change, there has been a global increase in the prevalence of intermittent rivers and the duration of their drying periods. The mutual inhibition mechanisms involving GHG emissions and the extended history of river drying has facilitated the restoration of ecosystem stability during rewetting.

1. There was a transformation of the biofilm from a carbon source to a carbon sink following prolonged drying, due to strong increases in the abundance of genes involved in the CBB cycle, and functional taxa such as *gemmatimonadota* and *pseudomonadota*.
2. *Gammaproteobacteria* serve as biomarker species across various experimental groups, playing a crucial role in the formation of biofilm ecological networks and significantly contributing to the emission of multiple GHGs.
3. Following cycles of wetting and drying, the availability of small-molecule carbon sources in the river has been found to be inadequate, further diminishing CO_2_ emissions during the decomposition of more complex carbon substrates.

## Supplementary Material

SI_ycaf187

Attachment_1_ycaf187

## Data Availability

The 16S and 18S sequence data that support the findings of this study are available in the NCBI repository (accession code: PRJNA1223359 and PRJNA1224600). The remaining data supporting the findings of this study are available within the article and source data file.
