# Supplementary material for: How river drying influences greenhouse gas emissions: insights from species and gene shifts
Source: ISME Commun. 2025 Oct 16;5(1):ycaf187. doi: 10.1093/ismeco/ycaf187 (PMC12596672; doi:10.1093/ismeco/ycaf187)
Supplement: SI_ycaf187 [file si_ycaf187.docx]

**How River Drying Influences Greenhouse Gas Emissions: Insights from Species and Gene shifts**

Chaoran Li^1^, Jun Hou^1^, Thibault Datry^2^, Tanveer M. Adyel^3^, Wei Zhou^4^, Jun Wu^1^, Guoxiang You ^1^, Tao Jin^5^, Ye Deng^6^, Lingzhan Miao^1*^

1Key Laboratory of Integrated Regulation and Resources Development on Shallow Lakes, Ministry of Education, College of Environment, Hohai University, 210098, Nanjing, People’s Republic of China

2INRAE, UR Riverly, Centre Lyon-Grenoble Auvergne-Rhône-Alpes, 5 Rue de La Doua CS70077, 69626, Villeurbanne Cedex, France

3Centre for Nature Positive Solutions, School of Science, RMIT University, VIC 3000, Melbourne, Australia

4 College of Geography and Remote Sensing, Hohai University, 210098, Nanjing, People’s Republic of China

5 Guangdong Magigene Biotechnol Co Ltd, Guangdong, People’s Republic of China

6 Key Laboratory of Environmental Biotechnology, Research Center for Eco-Environmental Sciences, Chinese Academy of Sciences, Beijing, People’s Republic of China

*Corresponding author:

Professor Lingzhan Miao

Author affiliation: Xikang Road 1^st^, Nanjing, People’s Republic of China, 210098

E-mail:[lzmiao@hhu.edu.cn](mailto:lzmiao@hhu.edu.cn);[mlz1988@126.com](mailto:mlz1988@126.com)

**Contents of this file**

Text S1 to S6

Figures S1 to S9

Tables S1 to S3

**Introduction**

[The following figures are mentioned in the manuscript as supplementary work to illustrate the main conclusions]

**Text S1 Biofilm culture and experimental design**

Biofilm colonisation was performed on the Qin Huai River (32°03 36.2 N; 118°44 38.1 E) in Nanjing, Eastern China, a tributary of the Yangtze River, which is under the control of the subtropical monsoon climate with annual average precipitation at 1038 mm (Figure S1 a-b). The water supply of this region’s run-off is mainly from the rainfall, which varies with the environment and is concentrated primarily in summer (June-August). Wire cages (30 cm in diameter and 30 cm in height) with cobbles (2–3 cm in diameter) were designed as biofilm colonisation devices (Figure S1 c). All incubation devices (wire cages with cobbles) were suspended 50 cm below the surface and attached to the shore railings by a fishing line to ensure the same flow conditions. Each device was cultured for 44 days under natural conditions to obtain mature biofilm on the cobbles [1, 2]. After 44 days of colonisation, all devices with cobbles and river water in the colonisation station were transferred to laboratory-flowing water channels (160 cm long, 20 cm wide, and 30 cm high) and divided into the control and three experimental groups with different drying gradients (20, 60 and 100 days) following three weeks of rewetting after drying (Fig S1 d and e). The channels were equipped with a pump (BT100-1L, Longer Precision Pump Co., Ltd., Baoding, China) to ensure water recycling and that the velocity of 0.14 m/s was maintained (consistent with the velocity of the Qin Huai River, Figure S1 d) [3]. Surface and free pore water in cobbles were drained from the experimental channels after flow cessation following the procedures in the previous study [4]. The biofilm was rewetting from small flows, avoiding destroying the biofilm on the surface of the cobbles. Then, the water column was filled to the control level using the pump [4]. Biofilms were prepared in quadruples for each sample test by randomly collecting specific amounts of cobbles in the channels. A sterile brush and tweezers were used to scrap the cobbles to collect the biofilms and wash the biofilm with 25 ml water in a ml centrifuge tube [3, 5].

**Text S2 Biofilm structure measurement**

**Water content and biomass**

Biofilm biomass was determined gravimetrically as dry weight (DW) and ash-free dry weight (AFDW) (Lu et al., 2016). A certain area (30 ± 2 cm^2^) of biofilms was scraped from cobbles and placed in a crucible. Samples were dried in an oven at 105 °C for 24 h to determine DW. Samples were ignited in a muffle furnace at 450°C for 5 h to determine AFDW. The ratio of AFDW to DW was calculated to measure the proportion of organic components.

**Biofilm cell viability analysis**

For the qualitative and quantitative determination of biofilm living and dead cells, the biofilm samples were initially placed on slides, washed with 0.85% NaCl buffer solution, and blotted with paper to remove excess water. Subsequently, the samples were stained with 200 μl of SYTO 9 (1:1000 dilution in purified water, Thermo Fisher Scientific) using a pipette and kept away from light for 15-30 minutes. Following the staining process, the samples were washed at least three times with 0.85% NaCl buffer solution until the stain was eluted and not fixed on the samples. They were then stored in 0.85% NaCl buffer solution. Next, 200 μl of propidium iodide (PI) (100 μl in 50 ml of 0.85% NaCl dilution, Bepo) was added to the samples, which were kept away from light for 15-30 minutes. After staining, the samples were again at least three times with 0.85% NaCl buffer solution until any unbound stain was removed and stored in 0.85% NaCl buffer solution. Finally, the samples were examined using confocal laser scanning microscopy (CLSM, Zeiss, Germany). A 485 nm laser was used to excite SYTO 9, producing green fluorescence for living cells, while a 536 nm laser was used to excite PI, producing red fluorescence for dead cells. Five regions were randomly selected from each sample for Z-stack image acquisition. Images were processed using Fiji software (ImageJ, NIH, USA) to differentiate between live and dead cells by setting thresholds and calculating ratios using plug-ins. All experiments were repeated at least three times, with data reported as mean ± standard deviation and subjected to analysis of variance (ANOVA), with p < 0.05 considered statistically significant.

**Spatial distribution of polysaccharides and proteins in biofilm**

To determine the spatial distribution of polysaccharides and proteins in the biofilm aggregates, the biofilm samples were first fixed at 4°C for 12 hours using 2.5% glutaraldehyde and washed three times with phosphate buffer solution (PBS). For protein staining, fluorescein isothiocyanate (FITC, 1 mg/ml DMSO or 70% ethanol dilution, Genye Bio) was used, which was excited at 488 nm to produce green fluorescence at 520 nm. For polysaccharide staining, Rhodamine-labelled concanavalin A (ConA, 100 μg/ml dilution in water, Thermo Fisher) was used to stain α-D-glucopyranose polysaccharides, excited at 561 nm to produce red fluorescence at 580 nm. β-D-glucopyranose polysaccharides were stained with a fluorescent brightener (CW, 1:10 dilution in water, Sigma) and excited at 400 nm to produce blue fluorescence at 435 nm. All staining treatments were incubated for 1 hour in the dark. A CLSM (Zeiss, Germany) was employed to capture the different fluorescence signals separately by setting the appropriate filter and laser parameters. Z-stack images were acquired from five randomly selected regions of the stained biofilm samples to fully analyse the spatial structure of the samples. Images were processed by Leica Application Suite X (LAS X) software, and thresholds were set appropriately to separate and quantify the signals of each fluorescence channel. Experimental data are presented in the form of images and quantitative analyses, and all experiments were repeated more than three times, with results reported as mean ± standard deviation, and ANOVA was performed using statistical software to determine the significance of differences between groups (p < 0.05).

**Text S3 Biofilm functions**

**Greenhouse gas emissions**

The CO_2_, N_2_O and CH_4_ emission rate are measured by placing cobbles covered with biofilm into a sealed chamber (20 cm in diameter and 20 cm in height), ensuring that the total biofilm area on the cobbles matches the chamber's bottom area. And pressure gauges are equipped on the top of the static chamber to detect changes in air pressure inside the static chamber. Water from an artificial stream is added to fully submerge the cobbles, and the chamber is sealed to prevent gas exchange with the environment. Air samples are drawn from the chamber's headspace every ten minutes and transferred to sealed bottles. These samples are then injected into a gas chromatograph (GC) to measure the concentrations of CO_2_, N_2_O and CH_4_. The GHG emission rate is calculated based on the change in GHG concentration over time, expressed in mg m^-2^ h^-1^, which represents the amount of GHG emitted per square meter of biofilm-covered surface area per hour. The flux was determined by a linear regression based on the change in CO_2_ (N_2_O and CH_4_) partial pressure (pCO_2_) (p N_2_O) (p CH_4_) over time [6, 7]. The CO_2_ (N_2_O/ CH_4_) flux (mg m^-2^h^-1^) was calculated according to Eq. (1), positive values represent emissions from the cobbles to the atmosphere, while negative values indicate an inflow from the atmosphere to the artificial stream channel [8]. This method allows for precise monitoring of GHG emissions, reflecting the metabolic activity of the biofilm under controlled conditions.

$F_{{CO}_{2}(N_{2}O/CH_{4})}=\rho*\frac{V}{A}\left( \frac{\Delta C({CO}_{2}/N_{2}O/CH_{4})}{\Delta t} \right)*(\frac{273}{T})$ (1)

where ${\Delta C({CO}_{2} /N_{2} O/CH_{4})}/{\Delta t}$ is the slope of the change in pCO_2_ (p N_2_O/p CH_4_) with time [μatm d^-1^], V is the volume of the chamber [m^3^], A is the surface area covered by the chamber [m^2^], T is the air temperature [K] and ρ is the ideal gas density.

**Gross Primary Production and Community Respiration**

The process begins with collecting cobbles placed in a natural river for biofilm culture and placing them evenly on the bottom of a sealed chamber (20 cm in diameter and 20 cm in height), ensuring that the total area of biofilm on the cobbles is approximately equal to the area of the chamber’s bottom. Water from an artificial stream is then added to the chamber, and a dissolved oxygen sensor (miniDO2T Logger, PME, USA) is placed in the water. The chamber is sealed, and the dissolved oxygen concentration is recorded every ten minutes for 120 minutes under constant light (to simulate daytime) and in the dark (to simulate nighttime). Community Respiration and Net Ecosystem Production (NEP) were measured from the drop and rise in dissolved oxygen concentration in chambers for 120 minutes in constant dark and light conditions (plant growth light; 1058mm×24mm×36.5mm; 18w; and 220v), respectively [9, 10]. Metabolic rates were calculated as described by Acuña and others [4, 11]. GPP and CR were calculated as Eq. (2-4).

$CR=\left( \frac{\Delta C_{treatment}}{\Delta t} \right)_{night}*\left( \frac{V_{water}}{A_{sed}} \right)$ (2)

$NEP=\left( \frac{\Delta C_{treatment}}{\Delta t} \right)_{day}*\left( \frac{V_{water}}{A_{sed}} \right)$ (3)

$GPP=NEP-CR$ (4)

Where C_treatment_ is oxygen (mg/L), t is time(min), night and day represent the dissolved O_2_ concentration data set used in the dark and light conditions (the measurement time is 120 min), V_water_ is the total water volume (L) in the metabolism chamber, and Ased is the measuring bed surface (estimated by the size and number of cobbles at the bottom of the chambers, m^2^).

**Carbon and nitrogen metabolism function**

The corresponding ELISA kit is a double antibody sandwich assay for the determination of the activity levels of various elemental metabolic enzymes in specimens. A solid phase antibody is made by coating a microtiter plate with purified metabolic enzyme (eg. α-D-Glu) antibodies, adding metabolic enzymes to the wells of the monoclonal antibody, and then combining them with the HRP-labelled metabolic enzyme antibody to form an antibody-antigen-enzymatic antibody complex. After thorough washing, the colour is developed by the addition of the substrate TMB, which is converted to blue by the enzyme HRP and to final yellow by the presence of acid. TMB is converted to blue by the enzyme HRP, and then to the final yellow colour by the action of acid. To prepare the standards for the assay, the kit provides an undiluted standard solution. Dilutions were performed in microtubes following a serial dilution scheme: 150 μl of undiluted standard was mixed with 150 μl of diluent to make the 200 U/L standard. This process was repeated down the line, each time using 150 μl of the previous dilution mixed with 150 μl of diluent, to prepare standards of 100 U/L, 50 U/L, 25 U/L, and 12.5 U/L. For sample addition, three types of wells were prepared on an enzyme-linked immunosorbent assay (ELISA) plate: blank wells with no sample or enzyme conjugate, standard wells with 50 μl of each standard, and test sample wells. In the test sample wells, 40 μl of sample diluent was added followed by 10 μl of the test sample, resulting in a 5x dilution. Samples were carefully added to the bottom of the wells to avoid touching the walls and were gently mixed by shaking. The plate was sealed with an adhesive film and incubated at 37°C for 30 minutes. Meanwhile, a 30x concentrated wash buffer was diluted to 1x using distilled water. After incubation, the sealing film was removed, the liquid was discarded, and the plate was flicked dry. Each well was filled with wash buffer, left for 30 seconds, and then emptied. This washing step was repeated five times, followed by tapping dry. 50 μl of enzyme conjugate was added to each well, except for the blank wells. The plate underwent a second incubation at 37°C for 30 minutes and was washed as described previously. For colour development, 50 μl of substrate solution A and 50 μl of substrate solution B were added to each well, mixed by gentle agitation, and incubated in the dark at 37°C for 10 minutes. The reaction was stopped by adding 50 μl of stop solution to each well, changing the solution colour from blue to yellow. Optical density (OD) at 450 nm was measured for each well using the blank wells to zero the plate reader. Measurements were taken within 15 minutes of adding the stop solution to ensure accuracy. The shade of colour correlates positively with the metabolic enzymes present in the sample. The absorbance (OD) was measured at 450nm using a Microplate Reader (Labsystems Multiskan MS 352, Finland) and the concentration of the metabolic enzyme activity of each element in the sample was calculated from the standard curve.

**Text S4**

**16S and 18S amplicon sequencing**

0.5 g biofilm samples were collected at every sampling time and stored at 80 ℃. The total DNA was extracted using a Magabio soil/fecal gfaecalc DNA purification kit (Bioer, Hangzhou, China). The quality and concentration of DNA were measured by Thermo NanoDrop One. For bacteria, the V4 hypervariable regions of the 16S rRNA gene were amplified using primer pair 515F(5`-GTGCCAGCMGCCGCGGTAA-3`) and 806R (5`-GGACTACHVGGGTWTCTAAT-3`) [12, 13]; for Eukaryote, the V4 region was amplified using 528F (5’ GCGGTAATTCCAGCTCCAA) and 706R (5’ AATCCRAGAATTTCACCTCT) primer sequences [14]. Amplicons were generated using specific primers with barcode and TaKaRa Premix Taq^®^ -Version 2.0 (TaKaRa Biotechnology Co., Dalian, China) and pooled. The thermal cycle conditions were as follows: 94℃ for 5min, 30 cycles of 94℃ for the 30s, 52℃ for 30s, 72℃ for 30s, and a final extension at 72℃ for 10min to complete the reaction, finally hold in 4℃. Four replicates were performed for each sample, and PCR products from the same sample were mixed by BioRad S1000 (Bio-rad Laboratory, CA).

After 1% agarose gel electrophoresis detection of PCR product fragment length and concentration, the concentration of PCR products was compared by GeneTools Analysis Software (Version 4.03.05.0, SynGene). The required volume of each sample was calculated according to the equal quality principle, and each PCR product was mixed. The PCR mixture was recovered using the E.Z.N.A.^®^ Gel Extraction Kit (Omega, USA) Gel Recovery Kit, and the target DNA fragment was eluted with TE buffer. Finally, the library was constructed according to the NEBNext^®^ Ultra^™^ II DNA Library Prep Kit for Illumina^®^ (New England Biolabs, USA) standard process, and PE250 sequencing was performed on the IlluminaHiseq2500 platform (Guangdong Magigene Biotechnology Co., Ltd. Guangzhou, China).

**Statistical analyses of sequencing data**

Sequenced paired-end Raw Reads were cut with sliding window quality (-W 4-M 20) using the fast (an ultra-fast all-in-one FASTQ preprocessor, version 0.14.1, <https://github.com/OpenGene/fastp>). Meanwhile, according to the fore and aft ends of the sequence, the primers were removed using cutadapt software (<https://github.com/marcelm/cutadapt/>) to obtain the quality paired-end Clean Reads. According to the relation between the overlap of PE reads, usearch- fastq_mergepairs (V10, <http://www.drive5.com/usearch/> preset parameters include the minimum length of overlap set as 16bp, the maximum error allowed in the overlap area of the overlapping sequence is 5bp, etc.) is used to filter disconfirmed tags, and Paired-end Clean Reads stitching is carried out to obtain the Raw Tags. Finally, fastq (an ultra-fast all-in-one FASTQ preprocessor, version 0.14.1, <https://github.com/OpenGene/fastp>) is used to cut Raw Tags by sliding window quality (-W 4-M 20) and obtain Clean Tags.

Operational taxonomic units (OTU) were clustered according to UPARSE ((RC Edgar. Highly accurate OTU sequences from microbial amplicon reads. DADA2[15] and Deblur [16] provided in QIIME2 (version 2020.11.0) process are used for denoising. Representative sequences for each OTU were taxonomically assigned by SILVA (16S/v132) and SILVA (18S/v132) Classifiers with a bootstrap threshold of 0.5 [17, 18]. Finally, singleton OTU, chimera, and contaminated OTU were removed to obtain each sample's effective sequence number of Tags (No. of seqs) and the comprehensive information table of OTU taxonomy (OTU_table).

**Text S5**

**Meta-genome sequencing**

Qualified DNA samples extracted during high-throughput sequencing were added to a fragmentation buffer and were subjected to random shearing using an ultrasonicator. The resulting short DNA fragments were then utilized for library construction. Each constructed library underwent quality control checks. Libraries that passed these checks were sequenced on the Illumina HiSeq 2500 high-throughput sequencing platform using PE150 sequencing. The raw image data obtained from sequencing was analyzed for base calling, and raw sequencing reads (Raw Reads) were converted into raw sequencing reads stored in FASTQ file format. This format included the sequence information of the reads and their corresponding quality information.

The bioinformatics analysis began with quality control, where the quality of the sequencing data was assessed, and low-quality data was removed to ensure the reliability of subsequent analysis. This step involved the use of the Trimmomatic software with parameters set to LEADING:3, TRAILING:3, SLIDINGWINDOW:5:20, and MINLEN:50. Post-quality control; clean reads were used for metagenomic assembly using MEGAHIT (v1.2.9, https://github.com/voutcn/megahit), specifying a minimum contig size of 500 bp. Open reading frames (ORFs) were predicted using Prodigal (https://github.com/hyattpd/Prodigal), and ORFs from assemblies were clustered into a non-redundant gene catalog with the parameters “-e 0.001 --min-seq-id 0.95 -c 0.8” using Linclust (https://www.nature.com/articles/s41467-018-04964-5). The abundance profile of non-redundant genes was estimated by mapping quality-trimmed reads from each sample against the gene catalogue (Unigenes). Species annotation of non-redundant Unigenes sequences was completed in BLASTP (Version 2.2.31+, http://blast.ncbi.nlm.nih.gov/Blast.cgi) by comparison with the NCBI-NR database (e-value<=0.0001). As a complement, the software Metaphlan was used to quickly classify Clean Reads to species. The predicted gene protein sequences were correlated with KEGG (diamond, e-value<=0.001), CAZyme (hmmscan, e-value<=1e-5), eggNOG (BLASTP, e-value<=0.001), BacMet & CARD (BLASTP, e-value<=0.0001), PHI (BLASTP, e-value<=1e-10), TCDB (BLASTP, e-value<=1e-20), VFDB (BLASTP, e-value<=1e-10) and other databases to obtain functional annotation information.

**Binning**

**Genome-resolved metagenomic analysis**

Of raw reads generated by llumina HiSeg and MiSeg sequencers, duplicates were removed as artifacts using an inhouse perl script, and unique reads were fltered to remove low quality bases/reads using Sickle (version 1.33) with the parameters “-q 20 -1 50” [19]. After that, all high-quality datasets were co-assembled using SPAdes (version 3.11.0) with the parameters “-k 21, 33, 55, 77, 99, 127 --meta”[20]. To calculate scaffold coverage, all high-quality reads from metagenomic datasets were mapped to the assembled scaffolds (length > 2000 bp)using BBMap with the parameters “minid =0.97, local=t”. These scaffolds were binned using MetaBAT (version 0.32.4) with the parameters “-m 2000 --unbinned"[21], which considers both tetranucleotide frequencies and the coverage of these scaffolds. The retrieved bins from MetaBAT were evaluated for taxonomic assignment, genome completeness, potential contamination, and strain heterogeneity, using CheckM [22], and were visualized using ESOM [23]. Afterwards, a cluster of four bins belonging to the Sva0485 group was further optimized to obtain high-quality genomes as previously described [24].

**Text S6**

**Microbial community network analysis**

Topological parameters were calculated following the equation (5-9), such as average degree (Avg K, indicating the species connectivity in the community [25, 26]), average path distance (GD, indicating the closeness of the network), Average clustering coefficient (AvgCC, indicating the extent of modular structure in the network) and Modularity (which is calculated to measure how well a network can be separated into modules) of networks. The visualisation of ecological networks also via Gephi (v0.9.2).

$k_{i}=\sum_{j\neq i} a_{ij}$ (5)

$AvgK=\frac{\sum_{i=1}^{n} k_{i}}{n}$ (6)

$GD=\frac{1}{n\left( n-1 \right)}\sum_{i\neq j} d_{ij}$ (7)

$CC_{i}=\frac{2l_{i}}{k_{i}^{'}\left( k_{i}^{'}-1 \right)}$ (8)

$Avgcc=\frac{\sum_{i=1}^{n} c_{i}c_{i}}{n}$ (9)

where $a_{ij}$ is the strength of the connection between nodes $i$ and $j$, $k_{i}$ is the degree of node i and $n$ is the number of all nodes; $d_{ij}$ is the shortest path between nodes $i$ and $j$; $l_{i}$ is the number of links between neighbouring nodes of node $i$ and $k_{i}^{'}$is the number of neighbouring nodes of node $i$.

In addition, the network stability was expressed by robustness which was calculated following the equation (10) [27, 28]. Robustness refers to a network`s vulnerability to random or targeted node removal, where the network is considered vulnerable when it breaks up into smaller parts as a result of node removal[29].

$w\mathrm{MI}S_{i}=\frac{\sum_{j\neq i} b_{j}s_{ij}}{\sum_{j\neq i} b_{j}}$ (10)

where$b_{j}$ is the relative abundance of species *j* and $s_{ij}$ is the strength of the Pearson correlation between species *i* and *j*. Thus $s_{ij}$ = $s_{ji}$. After deleting the selected node, if $w\mathrm{MI}S_{i}$ = 0 (all species links associated with *i* are deleted) or $w\mathrm{MI}S_{i}$ < 0 (there is no sufficient reciprocity between species *i* and other species), the node is considered extinct/isolated and therefore removed from the network removed. The proportion of remaining nodes was used as network robustness [28, 30].

**
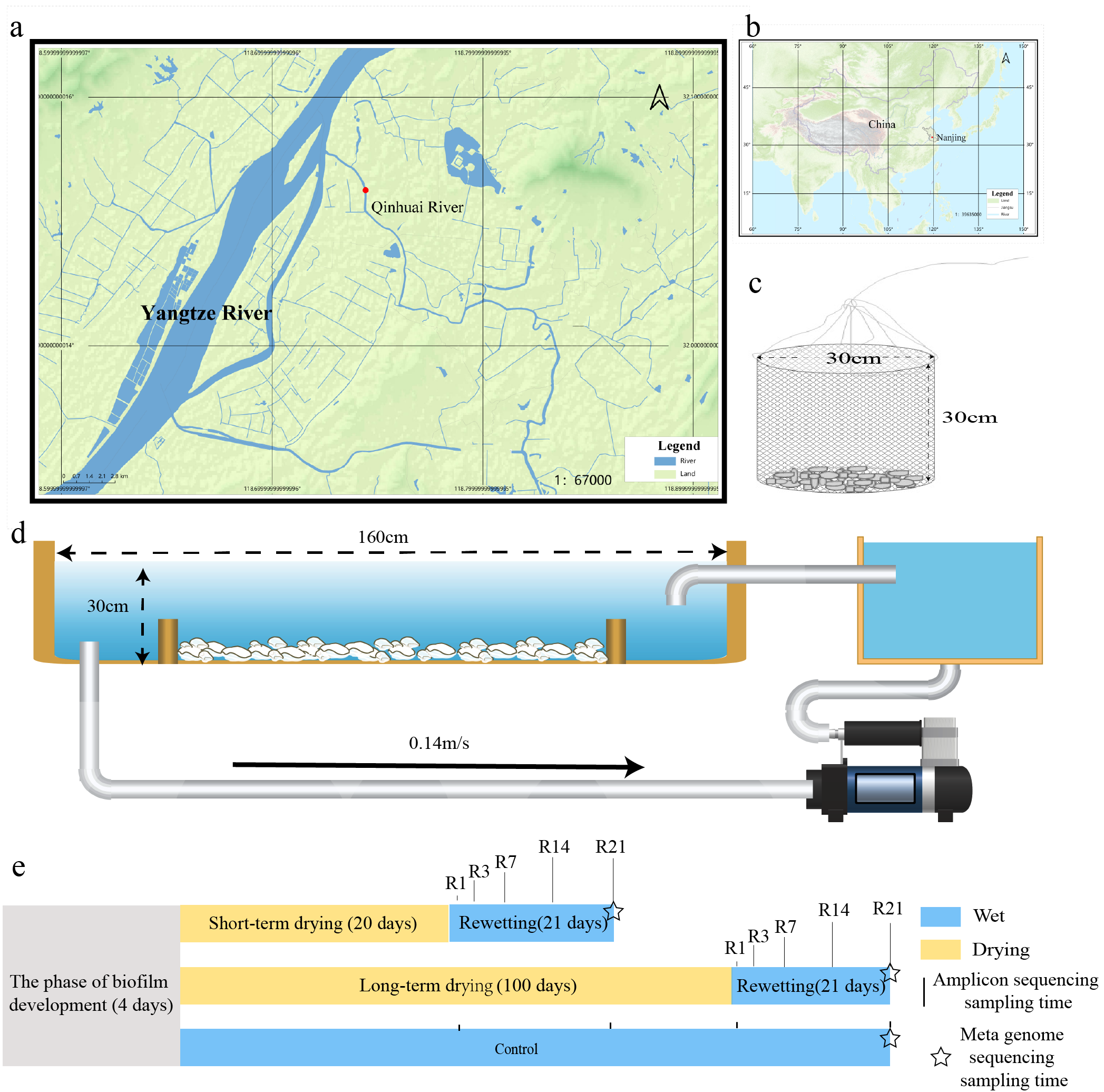
**

**Figure S1.** The location of biofilm culture (a and b), biofilm colonisation device (wire cages) (c), the schematic diagram of the experimental channel (d) and the experiment time axis of drying phase and wet alternation (e).

**
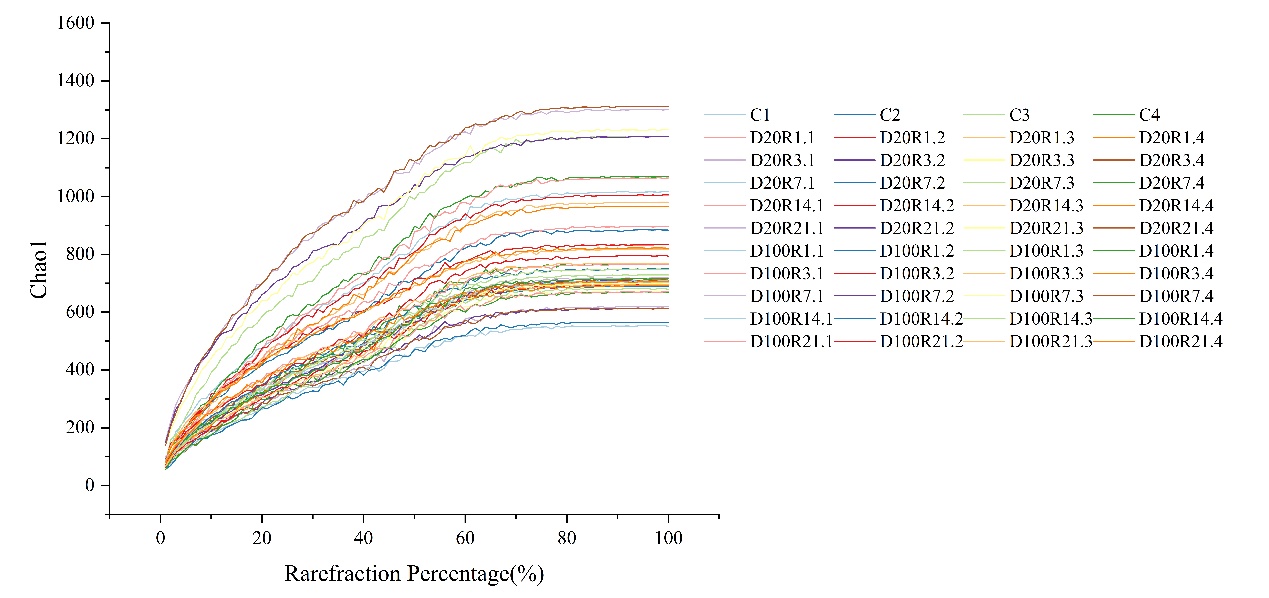
**

**Figure S2.** The rarefaction curve of bacterial Chao1 index at OTU level.

**
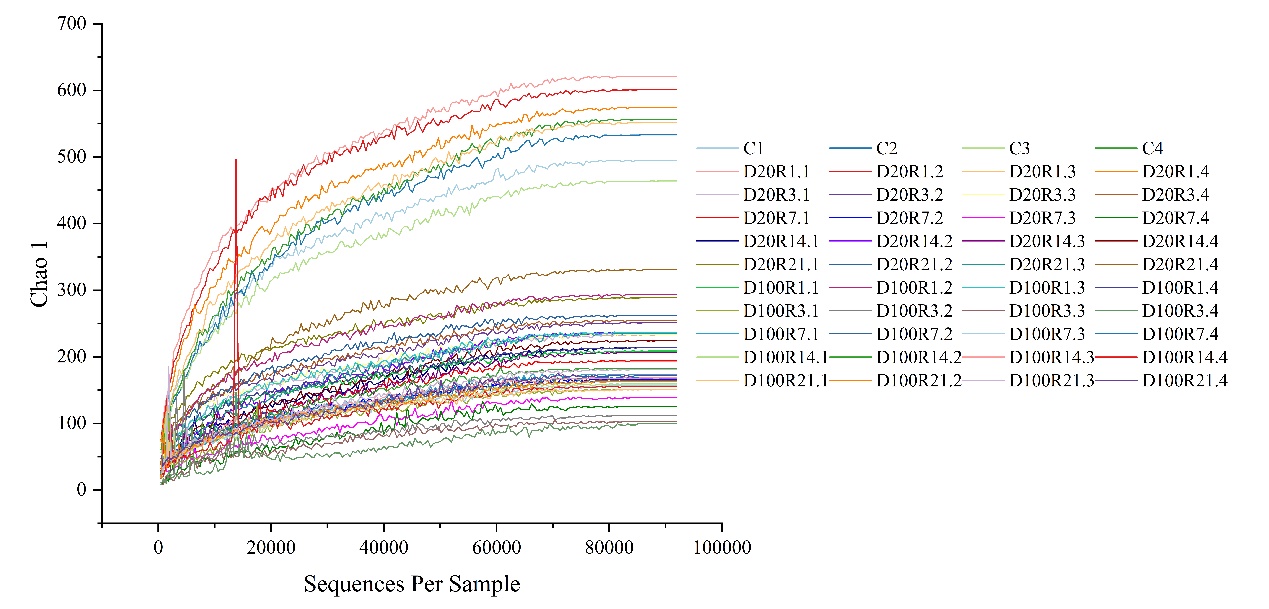
**

**Figure S3.** The rarefaction curve of eukaryotic Chao1 index at OTU level.


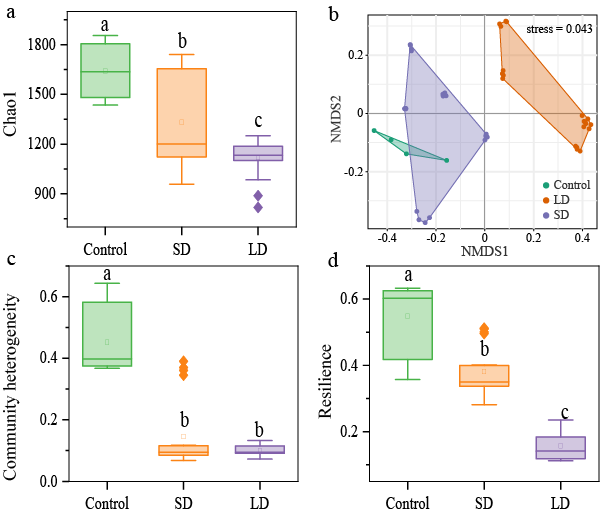


**Figure S4.** The biodiversity of biofilm at the OTU level during rewetting after different drying duration. SD: short-term drying; LD: long-term drying.

**
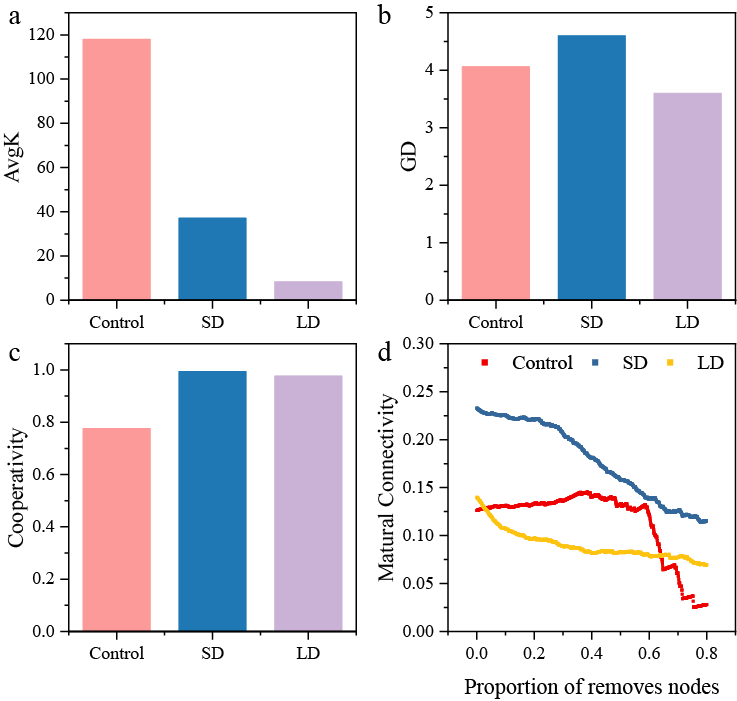
**

**Figure S5.** Topological properties of biofilm microbial networks. SD: short-term drying; LD: long-term drying.


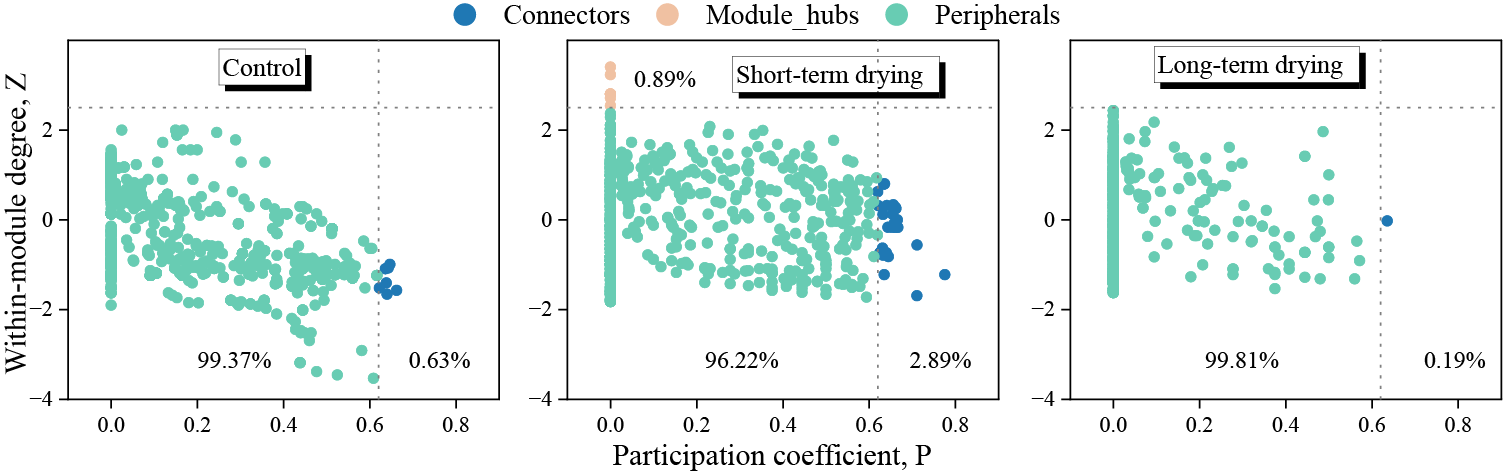


**Figure S6.** Zi-pi plot of microorganism species according to their network role. Each dot represents a species.

**
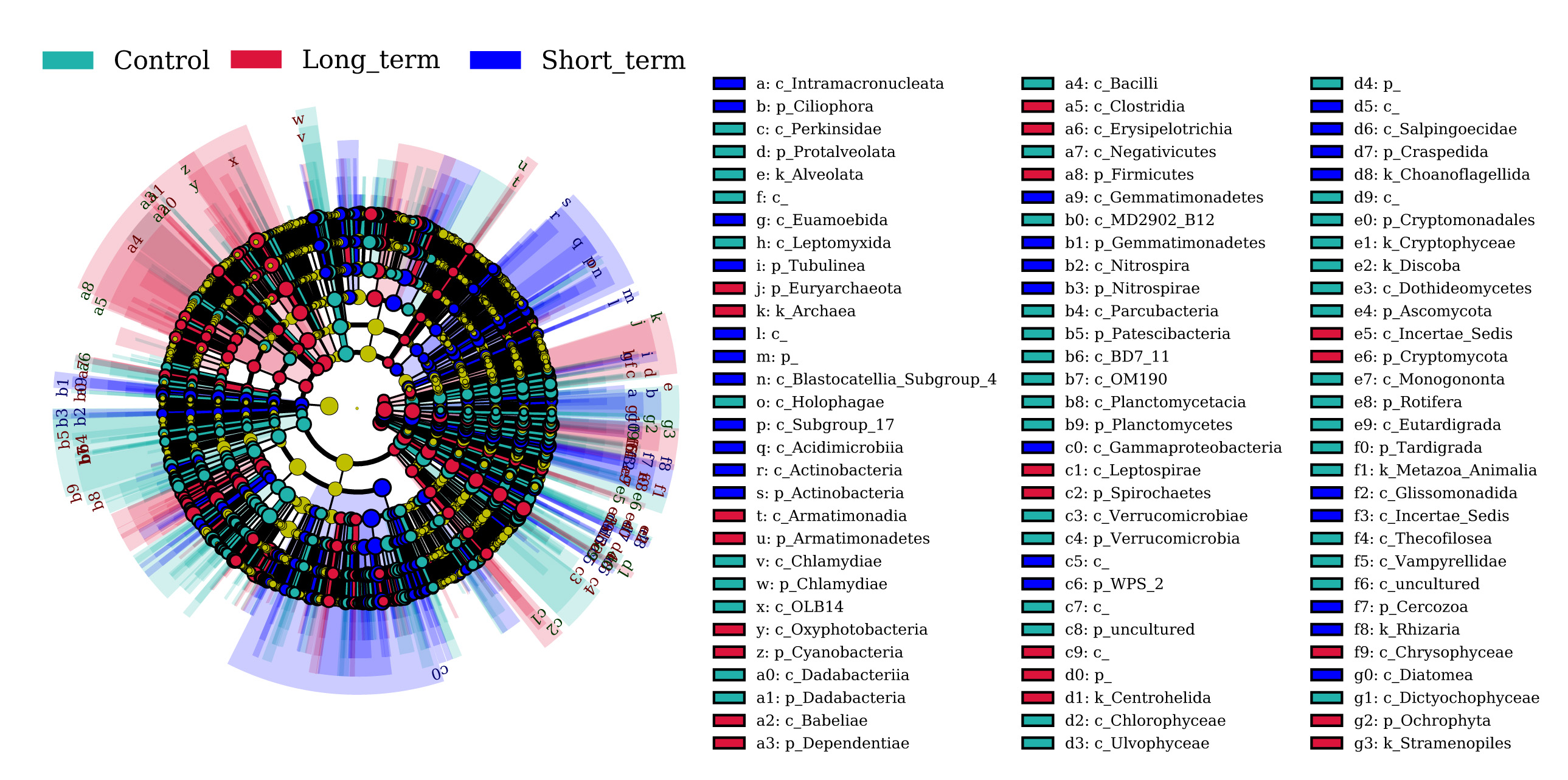
Figure S7.** Lefse results of biofilm microorganism.

**
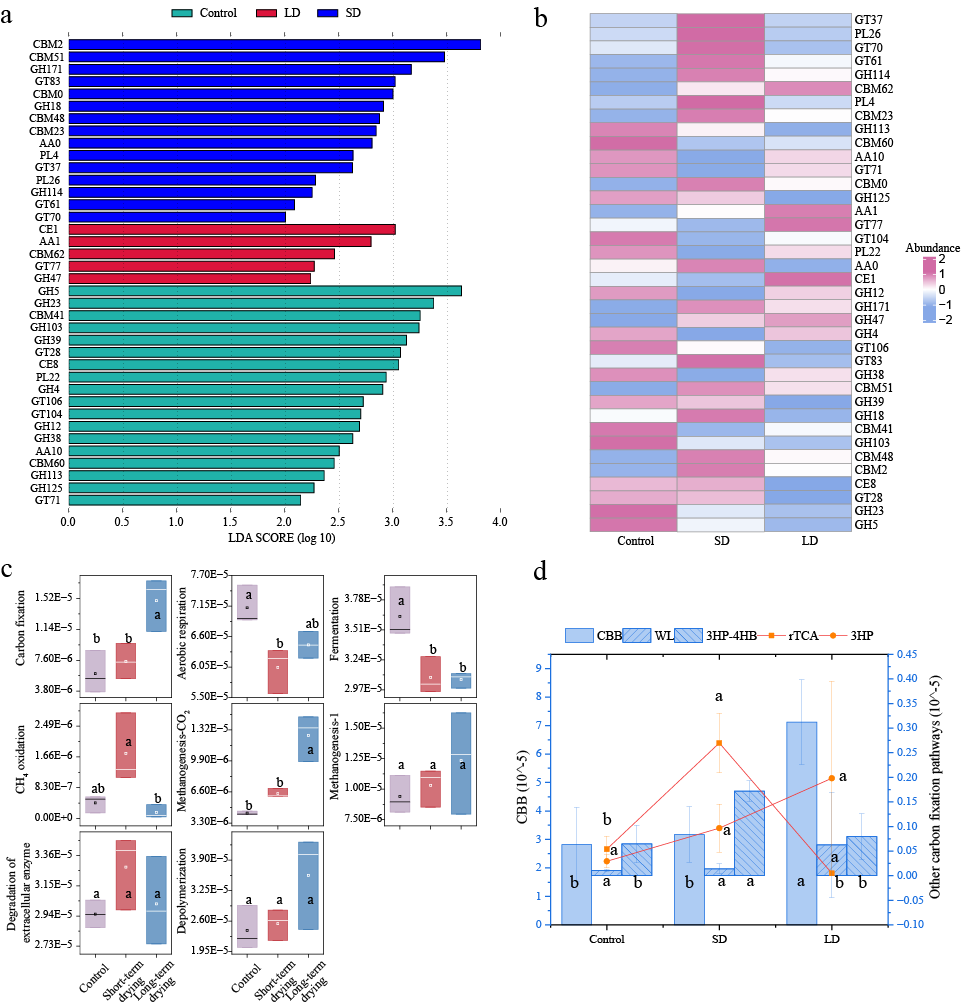
**

**Figure S8.** Variation of gene abundance in the biofilm carbon cycle. Biomarker gene abundance in different groups (a) and biomarker gene abundance (b), carbon cycle pathway gene abundance changes (c), six carbon fixation pathway gene abundance changes (d). SD: short-term drying; LD: long-term drying.


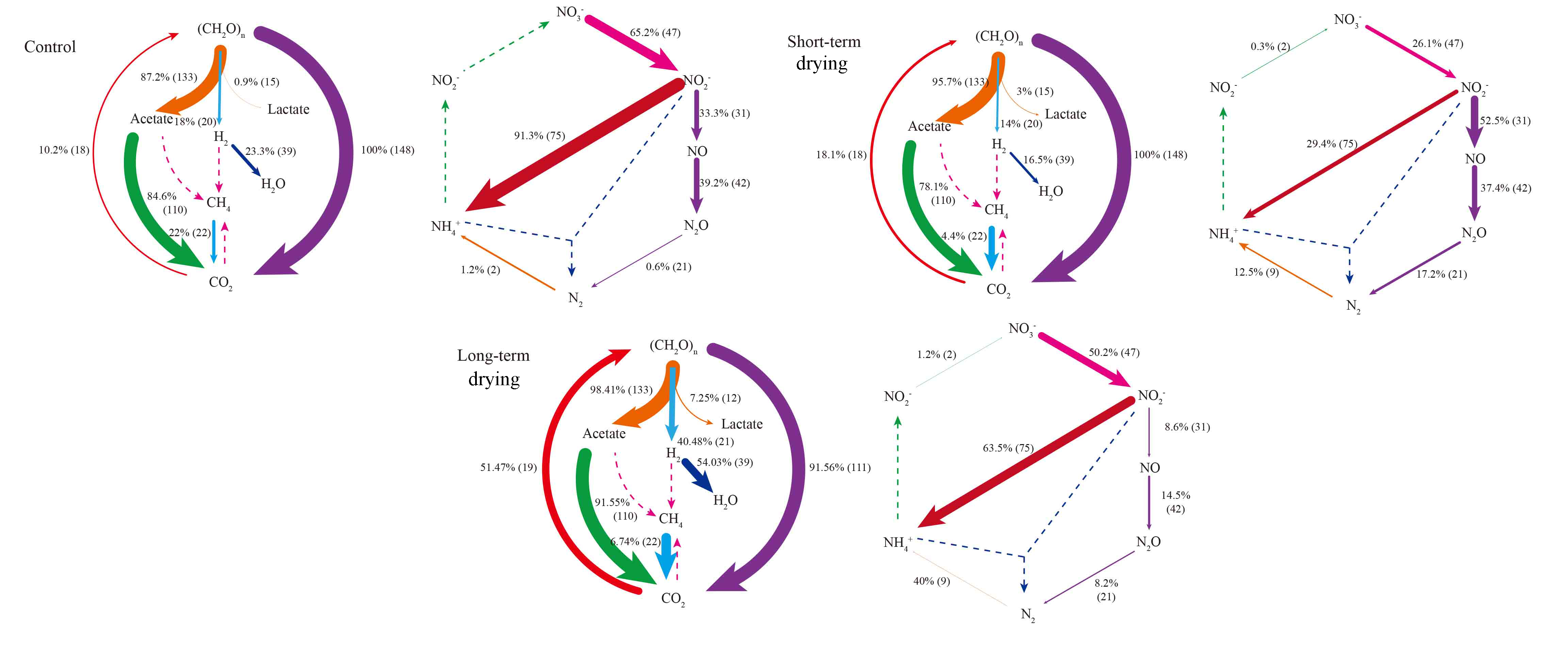


**Figure S9.** Changes in the proportion of active microorganisms along the carbon and nitrogen cycle path. The percentage refers to the percentage of the total microbial community that is on this path. The number of mags is in parentheses.

Table S1. Determination of the water qualities of the Qin Huai River.

| Water quality | Monitoring value |
| --- | --- |
| TN (mg/L) | 3.60±0.11 |
| $\mathrm{NH}_{3}^{-}-N$ (mg/L) | 2.93±0.02 |
| $\mathrm{NH}_{2}^{-}-N$ (mg/L) | 0.10±0.01 |
| TP (mg/L) | 0.11±0.01 |
| SS (mg/L) | 35.33±10.67 |
| Temperature (℃) | 18.40±0.06 |
| DO (mg/L) | 7.11±0.01 |
| pH | 7.63±0.28 |
| ORP (mV) | 314.20±2.95 |
| NTU | 20.91±0.82 |

Table S2. The main components of nutrient solution for the indoor adaptation stage.

| Component | | Concentration |
| --- | --- | --- |
| NaNO_3_ | | 8.51 g/L |
| CaCl_2_·2H_2_O | | 2.776 g/L |
| MgSO_4_·7H_2_O | | 3.697 g/L |
| NaHCO_3_ | | 1.26 g/L |
| Na_2_SiO_3_·9H_2_O | | 2.842 g/L |
| K_2_HPO_4_ | | 871 mg/L |
| H_3_BO_3_ | | 2.4 g/L |
| Microelement (WC) 25ml | Na_2_EDTA·2H_2_O | 109 mg/L |
|  | FeCl_3_·6H_2_O | 78.75 mg/L |
|  | CuSO_4_·5H_2_O | 62.5 μg/L |
|  | ZnSO_4_·7H_2_O | 550 μg/L |
|  | CoCl_2_·6H_2_O | 250 μg/L |
|  | MnCl_2_·4H_2_O | 4.5 mg/L |
|  | Na_2_MoO_4_·2H_2_O | 157.5 μg/L |
|  | Na_3_VO_4_ | 450 μg/L |
| Vitamin B12 | | 6.75 mg/L |
| Vitamin B1 | | 16.75 mg/L |
| Vitamin B7 | | 1.25 mg/L |

Table S3. The significant difference between different experimental groups of microbial community composition NMDS (PERMANOVA, p<0.01).

| Different experimental groups | Microbial community | |
| --- | --- | --- |
|  | R^2^ | Pr(>F) |
| Control vs Short-term Drying | 0.086201177 | 0.113 |
| Control vs Long-term Drying | 0.318447215 | 0.001 |
| Short-term Drying vs Long-term Drying | 0.392082176 | 0.001 |

**Reference:**

1. Battin, T.J., et al., *The ecology and biogeochemistry of stream biofilms.* Nature Reviews Microbiology, 2016. **14**(4): p. 251-263.

2. Wu, Y., et al., *In situ bioremediation of surface waters by periphytons.* Bioresource Technology, 2014. **151**: p. 367-372.

3. Liao, K., et al., *Integrating microbial biomass, composition and function to discern the level of anthropogenic activity in a river ecosystem.* Environment International, 2018. **116**: p. 147-155.

4. Zlatanovic, S., et al., *Shading and sediment structure effects on stream metabolism resistance and resilience to infrequent droughts.* Sci Total Environ, 2018. **621**: p. 1233-1242.

5. Liao, K., et al., *Use of convertible flow cells to simulate the impacts of anthropogenic activities on river biofilm bacterial communities.* Science of The Total Environment, 2019. **653**: p. 148-156.

6. Keller, P.S., et al., *Global CO2 emissions from dry inland waters share common drivers across ecosystems.* Nature Communications, 2020. **11**(1).

7. Gómez-Gener, L., et al., *When Water Vanishes: Magnitude and Regulation of Carbon Dioxide Emissions from Dry Temporary Streams.* Ecosystems, 2016. **19**(4): p. 710-723.

8. Campeau, A. and P.A. del Giorgio, *Patterns in CH4 and CO2 concentrations across boreal rivers: Major drivers and implications for fluvial greenhouse emissions under climate change scenarios.* Global Change Biology, 2014. **20**(4): p. 1075-1088.

9. Adyel, T.M., M.R. Hipsey, and C.E. Oldham, *Temporal dynamics of stormwater nutrient attenuation of an urban constructed wetland experiencing summer low flows and macrophyte senescence.* Ecological Engineering, 2017. **102**: p. 641-661.

10. Liu, K., et al., *Different community assembly mechanisms underlie similar biogeography of bacteria and microeukaryotes in Tibetan lakes.* FEMS Microbiol Ecol, 2020. **96**(6).

11. Acuña, V., et al., *Temperature dependence of stream benthic respiration in an Alpine river network under global warming.* Freshwater Biology, 2008. **53**(10): p. 2076-2088.

12. Caporaso, J.G., et al., *Global patterns of 16S rRNA diversity at a depth of millions of sequences per sample.* Proceedings of the National Academy of Sciences, 2011. **108**(supplement_1): p. 4516-4522.

13. Zhou, Y.Q., et al., *Warming reshaped the microbial hierarchical interactions.* Global Change Biology, 2021. **27**(24): p. 6331-6347.

14. Coleine, C., J.E. Stajich, and L. Selbmann, *Fungi are key players in extreme ecosystems.* Trends in Ecology & Evolution, 2022.

15. Callahan, B.J., et al., *DADA2: High-resolution sample inference from Illumina amplicon data.* Nature Methods, 2016. **13**(7): p. 581-+.

16. Amir, A., et al., *Deblur Rapidly Resolves Single-Nucleotide Community Sequence Patterns.* Msystems, 2017. **2**(2).

17. Vries, F.T.d., et al., *Soil bacterial networks are less stable under drought than fungal networks.* Nature Communications, 2018. **9**(1): p. 3033-3033.

18. Wang, Q., et al., *Naive Bayesian classifier for rapid assignment of rRNA sequences into the new bacterial taxonomy.* Applied and Environmental Microbiology, 2007. **73**(16): p. 5261-5267.

19. Joshi NA, F.J. *Sickle: A sliding-window, adaptive, quality-based trimming tool for FastQ files (Version 1.33) [Software].* 2011.

20. Bankevich, A., et al., *SPAdes: A New Genome Assembly Algorithm and Its Applications to Single-Cell Sequencing.* Journal of Computational Biology, 2012. **19**(5): p. 455-477.

21. Kang, D.D., et al., *MetaBAT, an efficient tool for accurately reconstructing single genomes from complex microbial communities.* Peerj, 2015. **3**.

22. Parks, D.H., et al., *CheckM: assessing the quality of microbial genomes recovered from isolates, single cells, and metagenomes.* Genome Research, 2015. **25**(7): p. 1043-1055.

23. Ultsch, A. and F. Morchen, *ESOM-Maps: tools for clustering, visualization, and classification with Emergent SOM*. 2009.

24. Chen, L.-X., et al., *Metabolic versatility of small archaea Micrarchaeota and Parvarchaeota.* The ISME Journal, 2018. **12**(3): p. 756-775.

25. Jiao, S., et al., *Stochastic community assembly decreases soil fungal richness in arid ecosystems.* Molecular Ecology, 2021. **30**(17): p. 4338-4348.

26. Wagg, C., et al., *Fungal-bacterial diversity and microbiome complexity predict ecosystem functioning.* Nature Communications, 2019. **10**.

27. Hu, W., et al., *Aridity-driven shift in biodiversity-soil multifunctionality relationships.* Nature Communications, 2021. **12**(1).

28. Montesinos-Navarro, A., et al., *Network structure embracing mutualism-antagonism continuums increases community robustness.* Nature Ecology & Evolution, 2017. **1**(11): p. 1661-1669.

29. Cohen, R., et al., *Resilience of the Internet to Random Breakdowns.* Physical Review Letters, 2000. **85**(21): p. 4626-4628.

30. Dunne, J.A., R.J. Williams, and N.D. Martinez, *Food-web structure and network theory: The role of connectance and size.* Proceedings of the National Academy of Sciences of the United States of America, 2002. **99**(20): p. 12917-12922.
